# Clinical characteristics of pediatric hospitalizations associated with 2009 pandemic influenza A (H1N1) in Northern Bavaria, Germany

**DOI:** 10.1186/1756-0500-5-304

**Published:** 2012-06-18

**Authors:** Anna Wieching, Jasmin Benser, Christina Kohlhauser-Vollmuth, Benedikt Weissbrich, Andrea Streng, Johannes G Liese

**Affiliations:** 1 University Children’s Hospital, Pediatric Infectiology and Immunology, Julius-Maximilians-University of Wuerzburg, Josef-Schneider-Str. 2, 97080, Wuerzburg, Germany; 2 Department of Pediatrics, Missio Hospital Wuerzburg, Salvatorstr. 7, 97067, Wuerzburg, Germany; 3 Institute for Virology and Immunobiology, Julius-Maximilians-University of Wuerzburg, Versbacherstr. 7, 97078, Wuerzburg, Germany

**Keywords:** Influenza, Pediatric, Infectious disease, Hospitalization

## Abstract

**Background:**

The 2009 pandemic influenza A (H1N1) (PIA) virus infected large parts of the pediatric population with a wide clinical spectrum and an initially unknown complication rate. The aims of our study were to define clinical characteristics and outcome of pandemic influenza A (H1N1) 2009-associated hospitalizations (PIAH) in children <18 years of age. All hospitalized cases of children <18 years of age with laboratory-confirmed pandemic influenza A (H1N1) 2009 in the region of Wuerzburg (Northern Bavaria, Germany) between July 2009 and March 2010 were identified. For these children a medical chart review was performed to determine their clinical characteristics and complications.

**Results:**

Between July 2009 and March 2010, 94 PIAH (62% males) occurred in children <18 years of age, with a median age of 7 years (IQR: 3–12 years). Underlying diseases and predisposing factors were documented in 40 (43%) children; obesity (n = 12, 30%), asthma (n = 10, 25%) and neurologic disorders (n = 8, 20%) were most frequently reported. Sixteen (17%) children received oxygen supplementation; three (3%) children required mechanical ventilation. Six (6%) children were admitted to an intensive care unit, four of them with underlying chronic diseases.

**Conclusions:**

Most PIAH demonstrated a benign course of disease. However, six children (6%) needed treatment at an intensive care unit for severe complications.

## Background

Influenza is a common cause of illness in children, predominantly treated as outpatients. For seasonal influenza, annual incidences for influenza-associated hospitalizations were estimated as 90 (CI 95%: 80–110) /100,000 in children <5 years old in the USA [1], and as 123/100,000 in children <6 years of age in a German region [2].

In 2009, an influenza pandemic was caused by a new influenza A/H1N1 virus (PIA). In more than 214 countries laboratory-confirmed PIA cases were reported, including over 18,097 deaths [3]. During this pandemic especially children appeared to be affected. In the USA, about 87,000 PIA hospitalizations (PIAH) and 1,280 fatalities occurred in children <18 years of age from April 2009 to April 2010, representing 32% of all PIAH and 10% of all PIA-associated fatalities in the US population [4].

In Germany, first cases of PIA were reported in April 2009; the first wave of the pandemic lasted from calendar week 42/2009 to 02/2010 [5]. The sentinel surveillance system for laboratory-confirmed PIA reported 226,137 cases and 253 fatalities for the total population until April 2010 [6]. A nationwide surveillance of critically ill children admitted to pediatric intensive care units (PICU) and fatalities associated with PIA estimated an incidence rate of 2.8 cases/100,000 children in infants <1 year of age and 0.8 cases/100,000 in children <15 years of age [7]. Thus far, there is only limited data on clinical characteristics and outcome of PIAH in Germany [8]. In the current study, we therefore investigated clinical characteristics of all pediatric PIAH in a defined geographical region.

## Methods

### Case definition and study population

All children and adolescents <18 years of age with laboratory-confirmed PIA admitted to one of the three hospitals covering pediatric in-patients in the area of Wuerzburg (Northern Bavaria / Germany) from July 2009 to March 2010, covering the influenza season 2009/2010, were included. The study population size in our defined catchment area corresponded to approximately 60,300 children younger than 18 years of age (Bavarian State Office for Statistics and Data Processing 2009, https://www.statistik.bayern.de).

### Laboratory methods

Respiratory samples (nasopharyngeal aspirate or nasopharyngeal swab or tracheal secretion) from all three hospitals were routinely sent to the Institute of Virology and Immunobiology of the University of Wuerzburg and analyzed using reverse transcriptase-polymerase-chain-reaction (RT-PCR) or direct immunofluorescence (DIF) testing for influenza A and B. All specimens tested positive for influenza by DIF were determined by PCR for subtype determination.

### Reporting system and data collection

Laboratory-confirmed PIA-infections were reported by the Institute of Virology and Immunobiology. For all identified PIAH patients <18 years of age a medical chart review was conducted by a representative of the study coordination centre (University Children’s Hospital, Wuerzburg). Date of admission, number of days in hospital, data on treatment, demographic, clinical and epidemiological data were obtained using a standardized and anonymous data collection form. All data were collected for the duration of hospital stay.

### Statistical analysis

For analysis of clinical parameters, all identified PIAH patients with symptoms starting before hospitalization or less than 72 h after hospitalization were included. Nosocomial infections with symptoms starting >72 h after hospitalization (n = 10 cases, including one fatality) were excluded from the present analysis and will be described elsewhere. Data were analyzed descriptively using SPSS (version 18.0 and 19.0, Chicago, IL). Continuous data were reported as median with interquartile range (IQR) and categorical variables as percentage of patients.

### Ethical review

The study was approved by the Institutional Ethics Review Board of the University of Wuerzburg.

## Results

**Figure 1:**
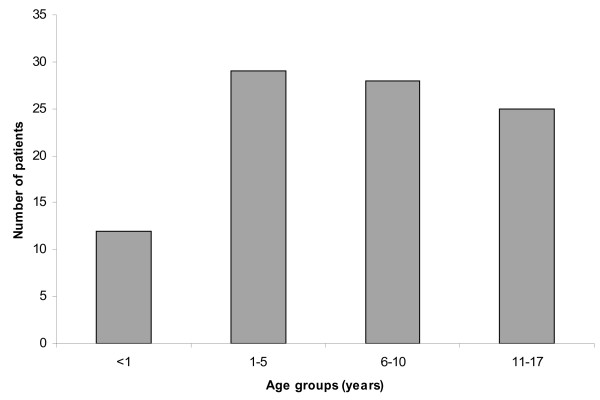
**Age distribution.** Age distribution of 94 hospitalized children with pandemic influenza A (H1N1) 2009, by age group.

**Figure 2:**
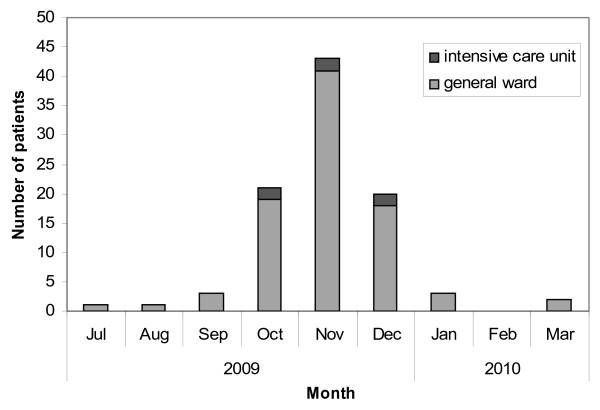
**Seasonal distribution.** Seasonal distribution of 94 hospitalized patients with pandemic influenza A (H1N1) 2009, during the pandemic influenza season 2009/2010.

A total of 94 (62% males) PIAH in children with a median age of 7 (IQR: 3–12) years were identified in the three hospitals between July 2009 and March 2010 (Figure 1). Seasonal distribution peaked in October to December 2009 with a maximum of 43 cases in November 2009 (Figure 2). Of all 94 patients, 88 (94%; 60% males, median 7 (2–12) years of age) were admitted to a general ward and stayed for a median of four (3–6) days in hospital. Six children (6%; 83% males, age range 0.1-16 years) were admitted to an intensive care unit (ICU), with a median stay at ICU of three (2–6) days and of seven (3–18) days in hospital. For 48 patients (51%), a contact with a person with suspected (n = 35) or confirmed (n = 29) PIA was reported as potential source of infection; most frequently, contacts to siblings (n = 30) or the child’s mother (n = 15) had been documented.

### Clinical presentation

The onset of symptoms, reported for 92 (98%) patients, occurred at a median of two (1–3) days before admission to hospital. The most frequently reported symptoms at admission were cough in 75 (80% of the 94 children), fever in 73 (78%), rhinorrhea in 48 (51%), and refusal of food or drink in 36 (38%) cases (Table 1). The most frequent diagnoses were upper respiratory tract infection in 84 (89%) children, bronchitis in 20 (21%; including nine (10%) cases of obstructive bronchitis), and pneumonia in 16 (17%) children, including one (1%) case of secondary bacterial pneumonia. Other reported diagnoses were fever convulsion in seven (7%), laryngitis/croup in six (6%), and otitis media in three (3%) children (multiple diseases per patient possible).

**Table 1:** Symptoms and diagnoses by age groups

	**Age groups (years)**			
	**<1 (n = 12)**	**1-5 (n = 29)**	**6-17 (n = 53)**	**<18 (n = 94)**
**Symptoms**				
Cough	8 (67%)	23 (79%)	44 (83%)	75 (80%)
Fever	10 (83%)	25 (86%)	38 (72%)	73 (78%)
Rhinorrhea	6 (50%)	18 (62%)	24 (45%)	48 (51%)
Food/drink refusal	6 (50%)	14 (48%)	16 (30%)	36 (38%)
Headache	0 (0%)	2 (7%)	22 (42%)	24 (26%)
Vomiting	0 (0%)	6 (21%)	15 (28%)	21 (22%)
Tachypnea	3 (25%)	2 (7%)	8 (15%)	13 (14%)
Conjunctivitis	1 (8%)	4 (14%)	5 (9%)	10 (11%)
Sore throat	0 (0%)	0 (0%)	9 (17%)	9 (10%)
Diarrhea	2 (17%)	3 (10%)	3 (6%)	8 (9%)
Myalgia	0 (0%)	1 (3%)	7 (13%)	8 (9%)
**Diagnoses**				
Upper airway infection	10 (83%)	26 (90%)	48 (91%)	84 (89%)
Bronchitis	5 (42%)	4 (14%)	11 (21%)	20 (21%)
- of these obstructive	5 (42%)	0 (0%)	4 (8%)	9 (10%)
Pneumonia	3 (25%)	6 (21%)	7 (13%)	16 (17%)
-of these secondary bacterial	0 (0%)	0 (0%)	1 (2%)	1 (1%)
Seizure	0 (0%)	4 (14%)	3 (6%)	7 (7%)
Laryngitis	1 (8%)	0 (0%)	5 (9%)	6 (6%)
Otitis media	2 (17%)	1 (3%)	0 (0%)	3 (3%)
Status asthmaticus	0 (0%)	0 (0%)	1 (2%)	1 (1%)
ARDS	0 (0%)	0 (0%)	1 (2%)	1 (1%)

Comparison of symptoms at presentation and diagnoses of 94 patients with pandemic influenza A (H1N1) 2009, by age group.

### Chronic conditions and predisposing factors

At least one underlying medical condition was documented for 40 (43%) of the 94 children; the most frequent condition was obesity defined as Body Mass Index >90th percentile (n = 12, 30% of all 40 children with predisposing factors), asthma (n = 10, 25%), neurologic disorders (n = 8, 20%), preterm birth, allergic diseases and other chronic diseases (each n = 6, 15%).

### Treatment

Oseltamivir was administered in 23 (25%) children for a median of three (2–5) days. A total of 28 (30%) children were treated with antibiotics, administered orally for a median of four (1–6) days and intravenously for a median of five (3–6) days. A total of 16 (17%) children received oxygen supplementation; three (3%) children required mechanical ventilation for a median of four (range 2–5) days.

### Patients admitted to an intensive care unit

ICU treatment was reported for six (6%) of the 94 patients. Four of them had known underlying chronic diseases or predisposing factors: *i)* A two-month-old former preterm (male) with congenital heart defect was admitted due to desaturation and pneumonia. After treatment with oxygen and antibiotics, he was discharged after three days on PICU and 31 days in hospital. *ii)* A ten-year-old boy with chronic neurologic and lung diseases was treated at PICU for one day due to fever convulsion. *iii*) A ten-year-old boy with tuberous sclerosis as chronic disease was admitted to PICU due to fever convulsion and was intubated and mechanically ventilated at the PICU for five days because of hypopnoea. *iiii)* A sixteen-year-old girl with obesity (BMI >30) and nicotine abuse as risk factors was treated at PICU due to pneumonia and acute respiratory distress syndrome. She was mechanically ventilated at the PICU for four days due to respiratory failure.

### Influenza vaccination status

Three of 94 hospitalized children (3%), two with underlying diseases, had been vaccinated against PIA five days (n = 2) or about two months before hospitalization (n = 1). One child without underlying diseases had been vaccinated against seasonal influenza before hospitalization (no information about date of vaccination). Only two (5%) of 40 children with underlying chronic conditions for whom influenza vaccination is generally recommended in Germany had received PIA vaccination, compared to one child (2%) of the 54 children without underlying chronic conditions. None of the six children with severe complications had received any influenza vaccination.

## Discussion

During the influenza A/H1N1 epidemic a total of 94 children with PCR-confirmed PIA were hospitalized in the three hospitals in Wuerzburg, Northern Bavaria, which cover the pediatric population of the city of Wuerzburg and its surroundings. The clinical course was mostly benign with cough (80%), fever (78%), and rhinorrhea (51%) as predominant symptoms. Only six percent of PIAH were admitted to an ICU; which is about three times lower than reported from Argentina, Canada and USA (17-19%) [9-11] but similar to 8% found in a UK hospital [12] and identical to 6% of ICU admissions reported from a large hospital in Hamburg (Northern Germany) [8]. In Germany, both in our study and in Hamburg, a clearly lower percentage of patients with PIA received treatment with oseltamivir (25% and 28%, respectively) and antibiotics (30% and 25%, respectively) [8], when compared to antiviral (46-99%) [9-11] and antibiotic (74-86%) treatment in other PIAH studies [9-11]. In Argentina, oxygen was supplemented five times more often (82%) than was to be observed in our study (17%) [11]. Mechanical ventilation was required six times more often (17%) than documented in our study (3%), whereas data from USA and Canada revealed a similar frequency (6%) for hospitalized patients [9,10]. The low rates of severe influenza cases in the present study correspond with the results of an earlier study on severe seasonal influenza in Germany [13]. Hence, on the one hand, the higher primary and secondary complication rates among hospitalized patients in other countries may reflect a real increase in the complication rate, due to delayed treatment with a limited access to primary health care. On the other hand, observed heterogeneity in the severity of hospitalized patients may result from differences in hospitalization access, with a higher threshold for hospitalization in countries with lower socio-economic status or limited health insurance (as in the USA) compared to Germany [11].

Underlying diseases were documented in 43% of PIAH, with asthma (25% of all children with predisposing factors) reported as one of the most frequent conditions. These results are comparable to the 32-40% of cases with underlying diseases (predominantly led by asthma) reported by most other surveys on pediatric PIAH [8,11,12,14]. Of 40 patients with underlying diseases, 10% received ICU treatment, in contrast to only 4% of 54 previously healthy patients, indicating a more severe course of disease in risk group children. However, the majority of children with underlying disease had an uncomplicated course of disease. It may be assumed that at least in part they were hospitalized for pre-emptive treatment and monitoring of possible complications. In contrast, a recent study from seven Austrian hospitals on PIA patients seeking emergency medical care reported underlying chronic conditions only in 13% of PIA patients <18 years of age [15].

In October 2009, the German Advisory Board on Immunization (STIKO) recommended vaccination against PIA for selected risk groups. For children, it was recommended that primarily children above six months of age with underlying diseases, such as chronic diseases of the air ways, cardiovascular system, liver or kidneys, should be vaccinated. Secondarily, healthy children should be vaccinated as well [16]. The first PIA vaccine was available in Germany at the end of October 2009. In our study, 40 (43%) children suffered from underlying diseases and, hence, ideally should have been vaccinated against influenza. Of these 40 children, two children were younger than six months and 16 children became ill before the PIA vaccine was available. Of the remaining 22 children with underlying diseases only two (9%) had received a PIA vaccination. Only for one child (2%) out of 51 children without predisposing factors aged above six months a PIA vaccination was reported. The low PIA vaccination coverage found in our study is confirmed by results from cross-sectional surveys in children <14 years of age in Germany (8% coverage) [17], and from a German surveillance study on severe PIA cases <15 years of age admitted to intensive care units (9% coverage) [7].

Potential limitations may result from the differences in criteria for inpatient treatment and use of diagnostic methods depending on the individual decision by the admitting physician. The number of hospitalizations corresponded to a conservative incidence estimate of at least 118 PIAH per 100,000 children <18 years of age. However, this may considerably underestimate the true pediatric PIAH incidence as only patients with laboratory-confirmed PIA were included; children hospitalized with respiratory symptoms or influenza-like illness without being tested for influenza were not captured in this study.

## Conclusions

The course of PIAH was predominantly benign; 43% occurred in children with chronic underlying diseases. Severe complications implying treatment at ICU occurred in six (6%) of the children, including four children with chronic underlying diseases. Better acceptance and higher vaccination coverage of risk group children with the recommended and available PIA vaccine could have prevented a considerable number of pediatric PIAH in Germany.

## Competing interests

JB, BW, AS and JL are currently conducting a separate investigator-initiated study on pediatric influenza which is financially supported by GlaxoSmithKline Biologicals (Rixensart, Belgium). JL and AS have also received a research grant for a previous investigator-initiated study on pediatric influenza by Novartis Vaccines (Marburg, Germany).

## Authors' contributions

AW and JB conducted the data collection and analyses in consultation with CK, BW, AS, JGL. AW and JB drafted the manuscript in consultation with CK, BW, AS and JGL. All authors read and approved the final manuscript.
